# Supplementary figures and images for: Large genomic deletions delineate Mycobacterium tuberculosis L4 sublineages in South American countries
Source: PLoS One. 2023 May 19;18(5):e0285417. doi: 10.1371/journal.pone.0285417 (PMC10198500; doi:10.1371/journal.pone.0285417)

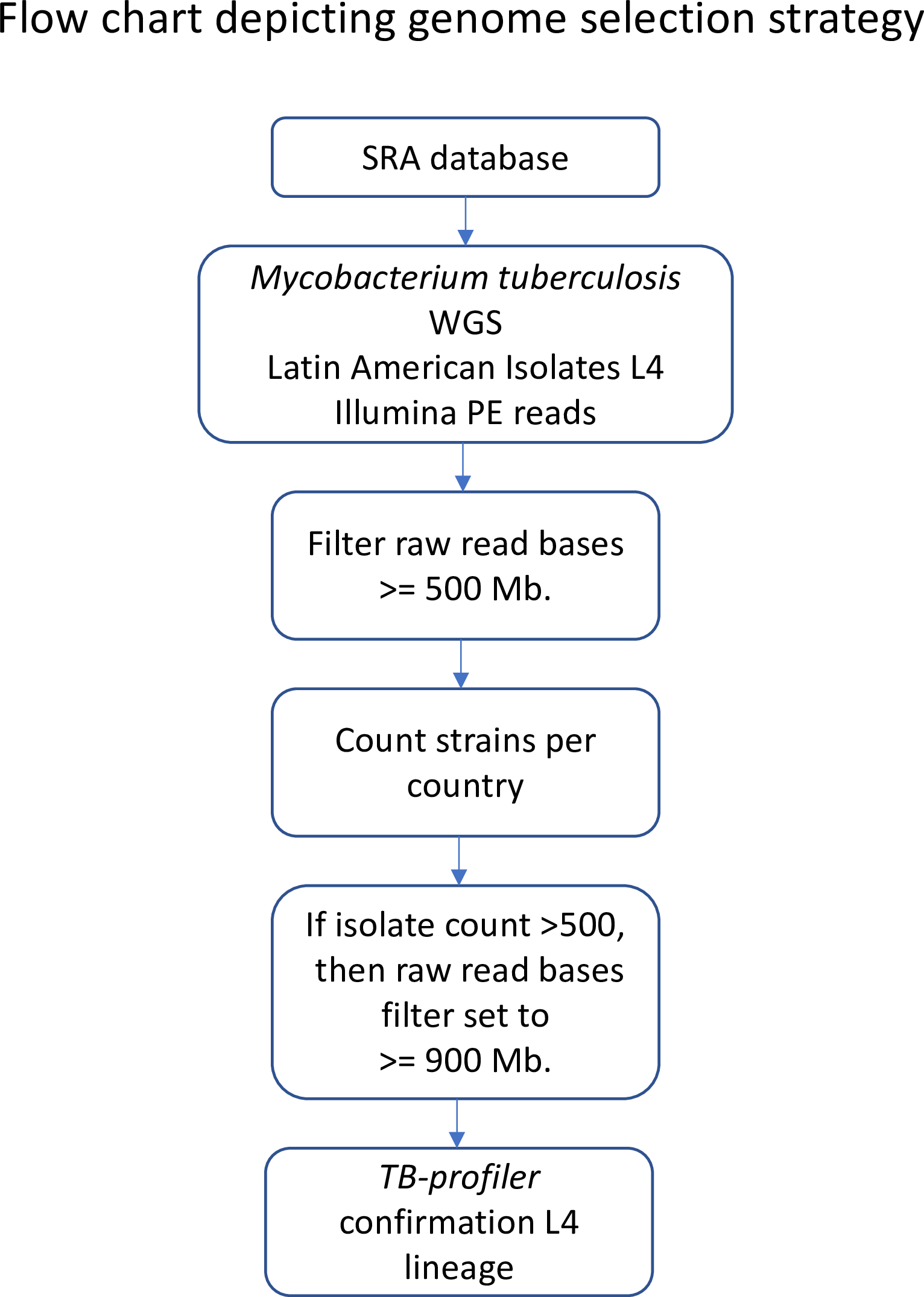

Supplement: S1 Fig — (TIF) [file pone.0285417.s001.tif]

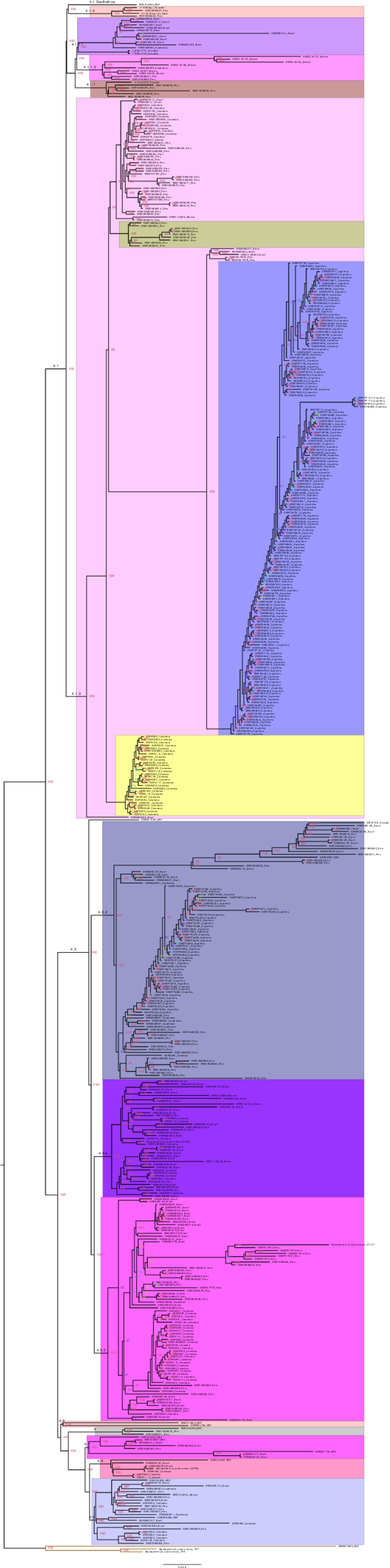

Supplement: S2 Fig — At the bottom, in brown lines, is the L2 outgroup. Numbers at nodes indicate ultrafast bootstrap support. Sublineages are labeled in their respective branches and highlighted with different colors. (TIF) [file pone.0285417.s002.tif]

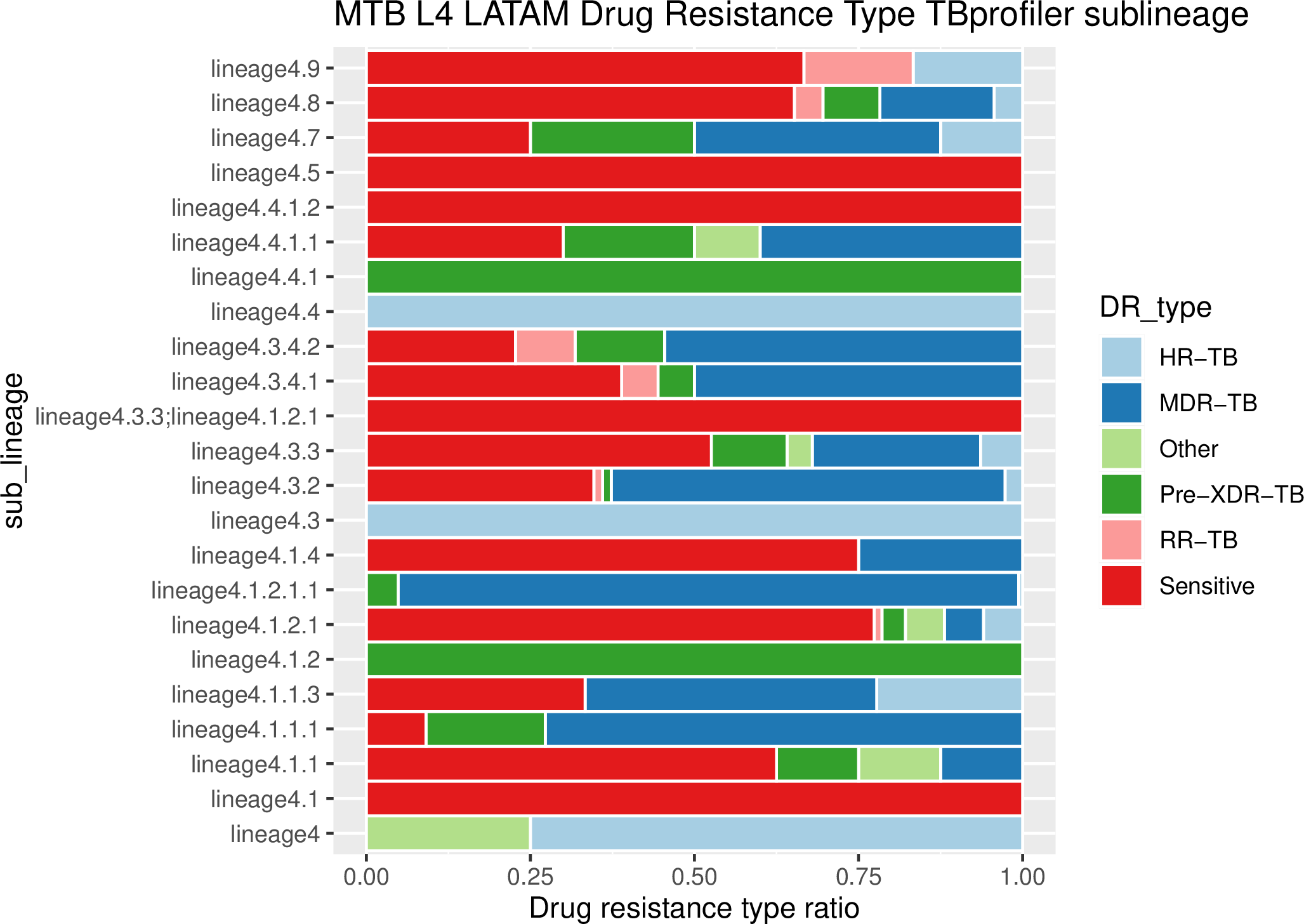

Supplement: S3 Fig — Drug-resistant types are depicted as normalized values on the X axis. (TIF) [file pone.0285417.s003.tif]
